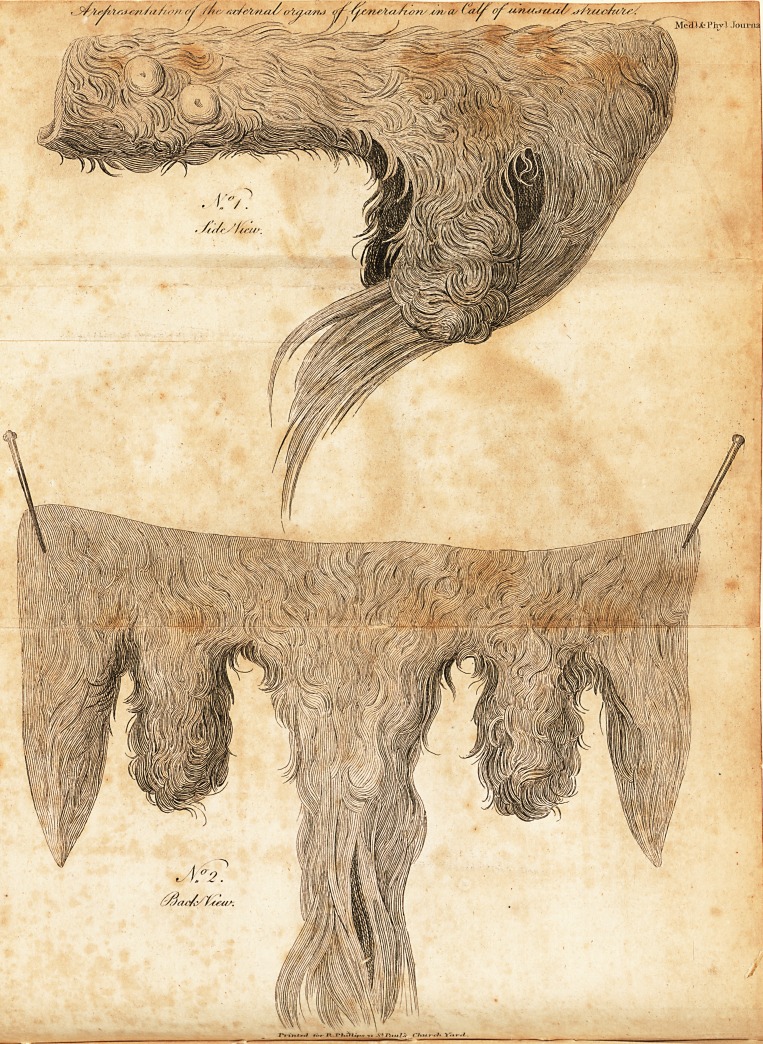# A Remarkable Structure and Appearance of the External Organs of Generation in a Calf

**Published:** 1799-11

**Authors:** W. Sandford

**Affiliations:** Surgeon


### Medical and Physical Journal.

VOL. II.] NOVEMBER, 1799. [NO. IX.

### *A remarkable Structure and Appearance of the External Organs of Generation in a Calf, with a Plate, communicated by* W. SANDFORD, *Surgeon.*

### *To the Editors of the Medical and Physical Journal.*

GENTLEMEN,

OBSERVING a Case of Lusus Naturæ, with a plate, in your Journal for August, it reminded me of one which I remarked about three years past, and of which I obtained a drawing. If you think it worth a place in your valuable Miscellany, it is at your service.

I am,

GENTLEMEN,

Your's, &c.

W. S.

*Worcester,*
*Sept.* 14, 1799.

TUESDAY, August 23, 1796, Mr. PAYNE, of Bridge-street, killed a calf ten weeks old, that was supposed to have in some respects the genital organs of both sexes united; and not being a twin calf, was on this account esteemed more remarkable.

On the first view the animal appeared to have testicles, which seemed disposed in a very singular manner, each testis having a compleat and separate scrotum, at a considerable distance from each other.

The penis, (as it afterwards proved to be) instead of proceeding in the usual direction along the abdomen of the calf, terminated in its sheath immediately between these apparent testicles, and in one point of view refembled the vagina of a cow, with its *bearing* rather more dependent than ufual.

This remarkable appearance feemed in fome degree confirmed by the animal voiding its urine backwards, in the fame manner as the cow.

Externally, and on each fide the abdomen, in their ufual fituation, were two teats or nipples, at the diftance of about three inches from the pouches or fcrota, as is fhewn in the drawing, No. 1.

In order to be fully fatisfied that the fubftance refembling the penis of the bull, was not an elongated *clitoris,** (a miftake that has fometimes happened,) I firft examined with a probe, and finding it pervious, introduced a director, and then laid it open to the ramus of the ifchium, and by this means traced the urethra along its courfe towards the bladder, as far as the inftrument could be paffed.

After this examination I diffected out the penis its whole length.

Thefe circumftances were more than fufficient to remove all doubt with refpect to its fituation and appearance, as an *elongated clitoris,* and at the fame time particularly to diftinguifh the fex.

The abdomen being then laid open, and the os-pubis carefully divided, I proceeded to examine the *internal* organs of generation, but found nothing in thefe by any means præternatural or confufed.

The teftes were within the abdomen, and were placed on each fide, correfponding to the pouches, † into which they would moft probably have defcended, had the calf been reared.

The diftance from the verge of the anus to the extremity of the fheath or vagina, meafured nearly *twelve inches.*

The appearances that may, perhaps, be efteemed moft worthy remark in the external genitals of this calf, are the fituation and diftinctnefs of the

* In fome inftances the penis has been miftaken for the clitoris, as was the cafe in a child of *feven years* old, upon whom, at *this period,* Mr. BRAND performed an *operation,* and by this means transformed an apparent girl into a boy; for a defcription of which fee "Cafe of a Boy miftaken for a Girl, with three Anatomical Views of the Parts, 4to printed for the author, *Soho-Square, London.* MURRAY, 1787."

† Thefe, upon examination, were difcovered to be formed of cellular and adipofe membrane.

the two fcrota, together with the fingularity in appearance, and deficiency in length, of the penis, from which circumftances (had the calf grown up) it would affuredly have been prevented from copulating, and at the fame time would have had the appearance of what is commonly called, an Hermaphrodite.

The *Free Martin*, (as it is ufually ftiled) has, I believe, the external parts of generation, refembling moft thofe of the common cow; the internal organs have been found to differ very materially. Thefe anatomical peculiarities, the late ingenious Mr. JOHN HUNTER * has given very accurate and fatisfactory defcriptions of, illuftrated by excellent engravings of the generative organs of three differently formed *Free Martins*, that he had an opportunity of diffecting.

The circumftance of the Free Martin *never* having been known to breed, is by fome experienced graziers faid to be *falfe*. Others have afferted, that the bull calf, under this defcription of twin, will not procreate unlefs he chance to be the firft twin calved; many other as extraordinary remarks have been made, that upon comparifon appear too vague and contradictory to place any dependence upon. This peculiarity, therefore, in the animal œconomy, and the degree of generative power annexed to it, muft remain in its prefent ftate of uncertainty till further experiments properly conducted, and refpectably authenticated, fhall be prefented to the public.

### EXPLANATION OF THE DRAWING, No. I.

TO have a perfect idea of the parts defcribed in this drawing, the fpectator muft imagine the leg and thigh on the left fide of the animal removed. In this view the fheath, together with the hairs towards its point, appear to take their natural direction, and are of the fame kind as thofe ufually obferved to grow upon this part when it terminates in its ufual fituation.

EXPLA-

* See his "Obfervations upon certain parts of the Animal Œconomy." 4to. p. 52.

In this Philofophical work, Mr. HUNTER has alfo given his ideas on the nature of Hermaphrodites; and remarks, that he has "frequently met with fheep, that appeared to be "*imperfect males, having the penis terminating in the perinæum, the orifice of which appeared like the bearing in the female.*" p. 51.

This was precifely the cafe in Mr. PAYNE's calf, and is therefore more reconcileable Mr. HUNTER's example of the *imperfect male* above defcribed, than of the Free Martin.

### EXPLANATION OF THE DRAWING, No. 2.

THIS exhibits the external parts when diffected from the abdomen and part of the perinæum; they are extended and reprefented in their pofition, to fhew the feparate formation of the two fcrota, and their diftance from each other, which, by meafurement when on the belly of the animal, was nearly fix inches.

---

## Figures and Tables

**No 1. No 2. f1:**